# Mitochondrial DNA Content Contributes to Climate Adaptation Using Chinese Populations as a Model

**DOI:** 10.1371/journal.pone.0079536

**Published:** 2013-11-08

**Authors:** Yao-Ting Cheng, Jia Liu, Li-Qin Yang, Chang Sun, Qing-Peng Kong

**Affiliations:** 1 State Key Laboratory of Genetic Resources and Evolution, Kunming Institute of Zoology, Chinese Academy of Sciences, Kunming, Yunnan, China; 2 KIZ/CUHK Joint Laboratory of Bioresources and Molecular Research of Common Diseases, Kunming, China; 3 Laboratory for Conservation and Utilization of Bio-Resources and Key Laboratory of Microbial Diversity in Southwest China, Ministry of Education, Yunnan University, Kunming, Yunnan, China; 4 University of Chinese Academy of Sciences, Beijing, China; University of Texas Health Science Center at San Antonio, United States of America

## Abstract

Maintaining a balance between ATP synthesis and heat generation is crucial for adapting to changes in climate. Variation in the mitochondrial DNA (mtDNA), which encodes 13 subunits of the respiratory chain complexes, may contribute to climate adaptation by regulating thermogenesis and the use of bioenergy. However, studies looking for a relationship between mtDNA haplogroups and climate have obtained mixed results, leaving unresolved the role of mtDNA in climate adaptation. Since mtDNA content can regulate human bioenergy processes and is known to influence many physiological traits and diseases, it is possible that mtDNA content contributes to climate adaptation in human populations. Here, we analyze the distribution of mtDNA content among 27 Chinese ethnic populations residing across China and find a significant association between mtDNA content and climate, with northern populations having significantly higher mtDNA content than southern populations. Functional studies have shown that high mtDNA content correlates with an increase in the expression of energy metabolism enzymes, which may accelerate thermogenesis. This suggests that the significantly higher mtDNA content observed in northern populations may confer a selective advantage in adapting to colder northern climates

## Introduction

Adapting to varied natural environments was key for the ancestors of modern humans to successfully settle the rest of world after they migrated out of Africa [1]. Indeed, such local adaptations have left a number of genetic imprints on the human genome [2] and shaped the distribution of the genetic variation among the human populations [3].

Climate is likely to have been an important selective pressure as humans moved to different latitudes and altitudes. The mitochondrion, an organelle crucial for energy production, and import in determining the balance between ATP synthesis and heat generation, is believed to have played an important role in environmental adaptation [4-7]. Accordingly, mitochondrial DNA (mtDNA), a small DNA molecule in the mitochondrion that encodes 13 subunits of the respiratory chain complexes [8], was suggested to have an important role in bioenergy production? and thermogenesis and thus in climate adaptation in human populations [9,10]. By analyzing a large number of complete mtDNA sequences from different human populations, some researchers suggested that the contemporary distribution of mtDNA lineages or haplogroups (a group of haplotypes that share common mutations and a most recent common ancestor) was the result of climate adaptation [9,10]. However, this view was not supported by subsequent studies [11-14], and the effect of climate on the distribution of mtDNA lineages remains an open question.

Remarkably, mtDNA copy number is an important factor in regulating human bioenergy processes such as the balance between ATP production and thermogenesis [15]. Consequently, variation in mtDNA content between individuals or across time could contribute to many physiological traits and diseases, such as aging and cancer [16-19]. These observations raise the intriguing possibility that mtDNA content, rather than mtDNA haplogroup, may contribute to the adaptation of human populations to different environments. To investigate the distribution of mtDNA content among different Chinese ethnic populations and its relationship with climate, we collected and measured the mtDNA content from a total of 1207 Chinese individuals, representing 27 ethnic populations residing in the different regions of China. Our results show that increased mtDNA content is associated with a decline in environmental temperature. Taking into account the importance of mtDNA content in bioenergy and thermogenesis regulation, our study suggests that mtDNA content may contribute to human adaptation to different environments, at least in Chinese populations.

## Results and Discussion

As shown in Table 1, the total mean relative value of the mtDNA content (the ratio of mtDNA to nuclear DNA, also referred to as the mt/n ratio) is 41.23, and the range of mean mtDNA content varies across populations from 19.30 to 66.33. Similar to previous observations [20-22], mtDNA content varies among different Chinese ethnic groups. Hence, to avoid spurious results, extreme caution should be taken when choosing control samples in any the future association studies by using mtDNA content.

**Table 1:** Sampling information.

**Population ID**	**Number**		**Mean mtDNA content**	**AAAP^a^ (0.1hPa)**	**AAT^b^ (0.1°C)**	**AATmax^c^ (0.1°C)**	**AATmin^d^ (0.1°C)**	**AARH^e^ (-0.1)**	**ASH^f^ (0.1h)**	**Weather Station ID**	**Latitude**	**Longitude**	**Sample Location**	**Region**
Hui_IM	56	51.35		9274	-44	42	-124	72	24966	50434	50.48	121.68	Argun, Inner Mongolia	NE^g^
Buryat	44	51.7		9419	-10	55	-67	68	27188	50527	49.21	119.72	Ewenki Autonomous Banner, Inner Mongolia	NE
Ewenki	22	46.41		9263	-4	58	-62	64	27202	50632	49.02	123.27	Hulun Buir, Inner Mongolia	NE
Mongolian_IM	55	50.59		8501	28	92	-26	56	30767	53391	43.92	115.99	Boarder Yellow Banner, Inner Mongolia	NE
Daur	20	51.49		9966	39	99	-15	60	28392	50745	46.41	121.88	Ewenki Autonomous Banner, Bayan Tal, Inner Mongolia	NE
Xibe_IM	24	51.77		9966	39	99	-15	60	28392	50745	48.12	123.46	Arun Banner, Inner Mongolia	NE
Mongolian_QH	12	43.32		7719	61	138	4	55	26756	52866	36.62	101.76	Xining, Qinghai	QT^j^
Han_JL	37	31.53		9959	67	125	15	65	26834	54157	43.16	124.35	Siping, Jilin	NE
Mongolian_XJ_BZ	43	34.86		9121	69	126	22	58	25233	51463	43.82	87.61	Mongolian Autonomous Prefecture of Bayingolin, Xinjiang	NW^h^
Mongolian_XJ_BL	39	46.88		9834	78	142	22	62	25540	51334	44.88	82.07	Bole, Xinjiang	NW
Mongolian_XJ_YL	61	34.41		9418	90	163	25	65	28521	51431	43.94	81.47	Yili, Xinjiang	NW
Xibe_XJ	115	37.88		9418	90	163	25	65	28521	51431	43.94	81.47	Yili, Xinjiang	NW
Uighur_N	24	39.78		9418	90	163	25	65	28521	51431	43.94	81.47	Yili, Xinjiang	NW
Hui_GS	130	33.72		8482	98	167	45	56	24240	52889	36.06	103.8	Linxia, Gansu	NW
Korean	28	32.87		10154	89	140	49	69	24590	54497	40.13	124.38	Dandong, Liaoning	NE
Uighur_S_HT	31	29.88		8623	125	191	68	43	25870	51828	37.11	79.92	Hetian, Xinjiang	NW
Han_SX	38	59.69		9703	137	193	92	70	16461	57036	34.26	108.93	Xi'an, Shannxi	NW
Han_JS	63	43.77		10123	145	197	100	69	22209	58027	33.6	119.02	Huai'an, Jiangsu	S^i^
Han_YN	40	44.5		8106	149	208	103	73	21976	56778	25.04	102.71	Kunming, Yunnan	S
Tujia	27	19.3		9837	165	211	132	80	14819	57745	27.43	109.69	Fenghuang, Hunan	S
Dai	41	38		8685	187	246	146	72	21615	56985	23.36	103.36	Mengzi, Yunnan	S
Han_GD	34	22.26		10131	215	252	187	81	19787	59316	23.35	116.67	Shantou, Guangdong	S
Oroqen	34	66.33		9842	4	70	-60	66	27260	50557	50.48	121.68	Oroqen Autonomous Banner, Inner Mongolia	NE
Kazak	61	45.14		8720	118	183	55	52	27264	51709	39.5	76.04	Kashi, Xinjiang	NW
Kirgiz	51	41.66		8720	118	183	55	52	27264	51709	39.5	76.04	Kashi, Xinjiang	NW
Maonan	34	41.29		9887	205	249	176	76	12585	59023	24.69	108.08	Hechi, Guangxi	S
Mulam	43	39.56		9887	205	249	176	76	12585	59023	24.69	108.08	Hechi, Guangxi	S

a. AAAP, annual average atmospheric pressure; b. AAT, the annual average temperature; c. AATmax, the average maximum temperature; d. AATmin, the average minimum temperature; e. AARH, annual average relative humidity; f. ASH, annual sunshine hour; g. NE, northeastern China; h. NW, northwestern China; i. S, southern China; j. QT, Qinghai-Tibet plateau of China.

**Figure 1:**
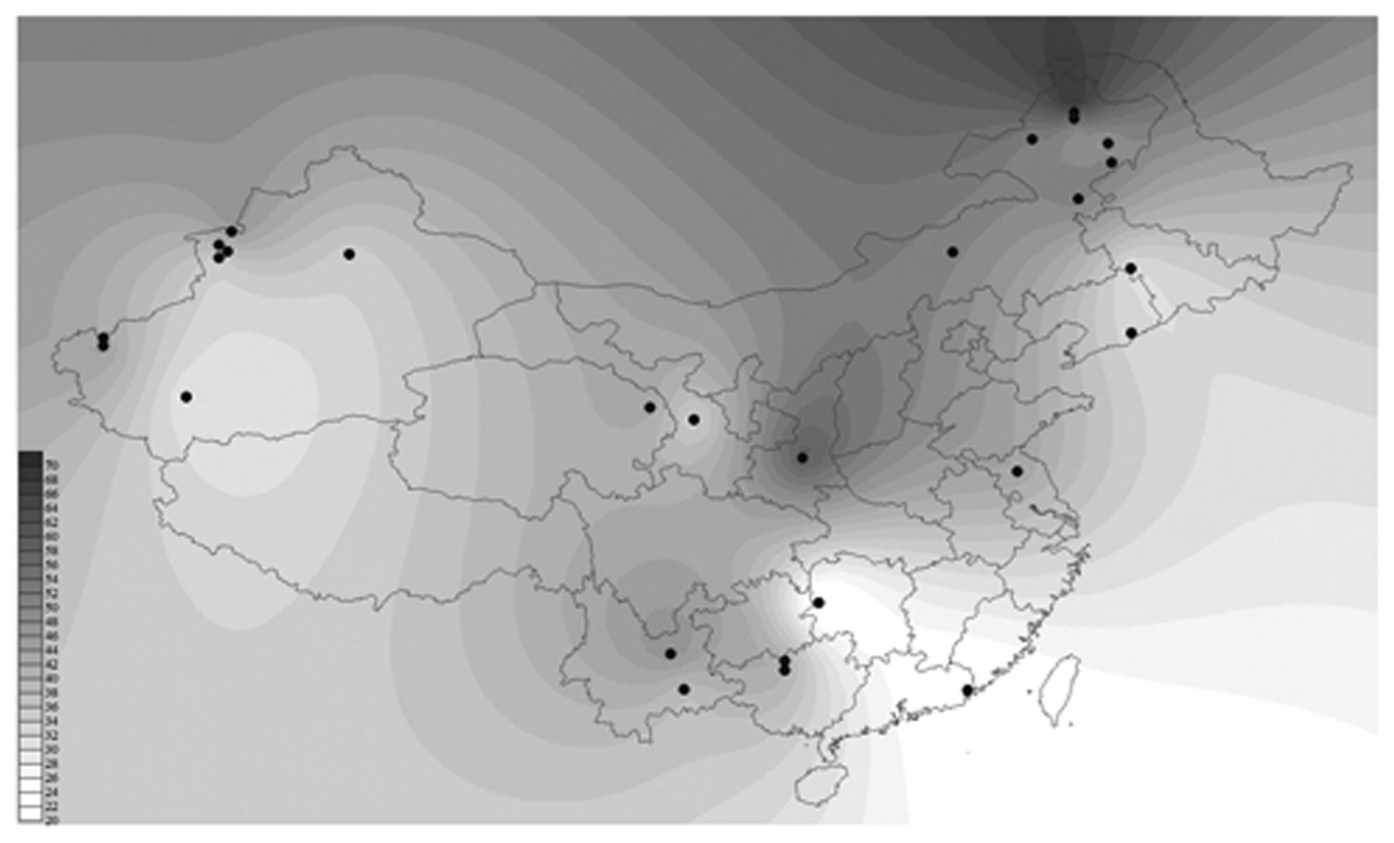
mtDNA content in Chinese populations. The mtDNA content of the northern populations is significant higher than that of the southern populations (*P*_*N/S*_=0.1×10^-2^), with the highest mean content observed in the northeastern populations (NE) and the lowest in the southern populations (*P*_*NE/S*_=6.7×10^-8^, *P*_*NW/S*_=0.23). Darker colors represents higher levels of mtDNA content.

**Figure 2:**
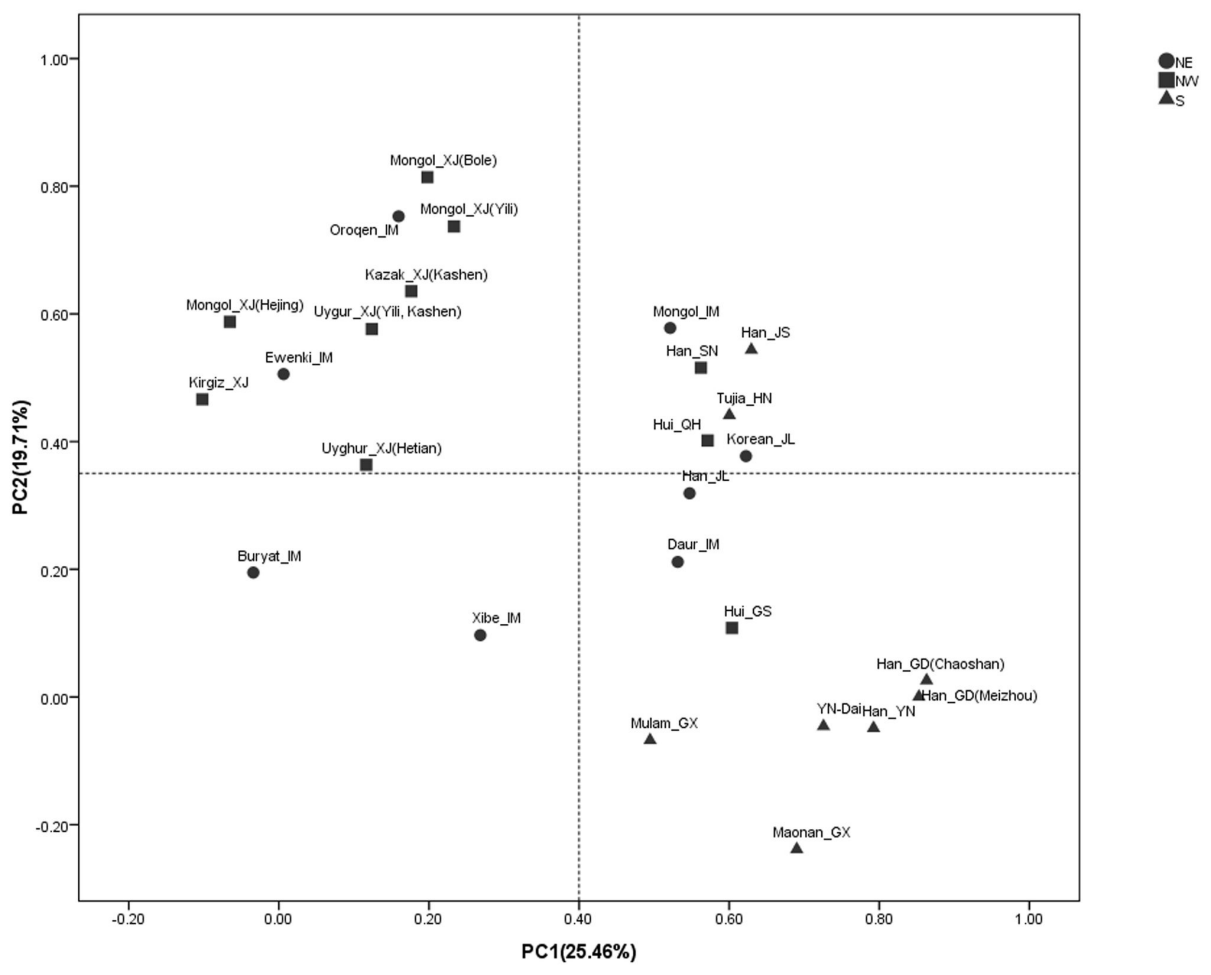
Principal components analysis of mtDNA haplotypes in the 26 populations. The analysis was performed using the basal haplogroup frequency matrix for the 26 Chinese populations retrieved from the literature (Table S2).

Further analysis revealed a declining gradient of mtDNA content from north to south in Chinese populations. Specifically, the mtDNA content of the northern (N) populations is significantly higher than that of the southern (S) populations (*P*_*N/S*_=1×10^-3^), with the highest mean value in the northeastern populations (NE) and the lowest in the southern populations (*P*_*NE/S*_=6.7×10^-8^, *P*_*NW/S*_=0.23) (Figure 1). To test whether the observed distribution pattern of mtDNA content is shaped by the mtDNA lineages and in fact a reflection of the distribution of mtDNA haplogroups, we collected published mtDNA sequence data from the 26 Chinese populations considered in this study [20-28]. The results obtained from principal component analysis (PCA) and canonical correlation analyses revealed that there is no significant correlation between the haplogroup distribution and variation in mtDNA content (canonical correlation analysis; *P*=0.36; Figure 2), suggesting that haplogroups have little influence on the variation in mtDNA content. Therefore, it seems unlikely that the contemporary distribution of mtDNA content among the populations is affected by haplogroups and thus the ethnic origins of the populations.

**Figure 3:**
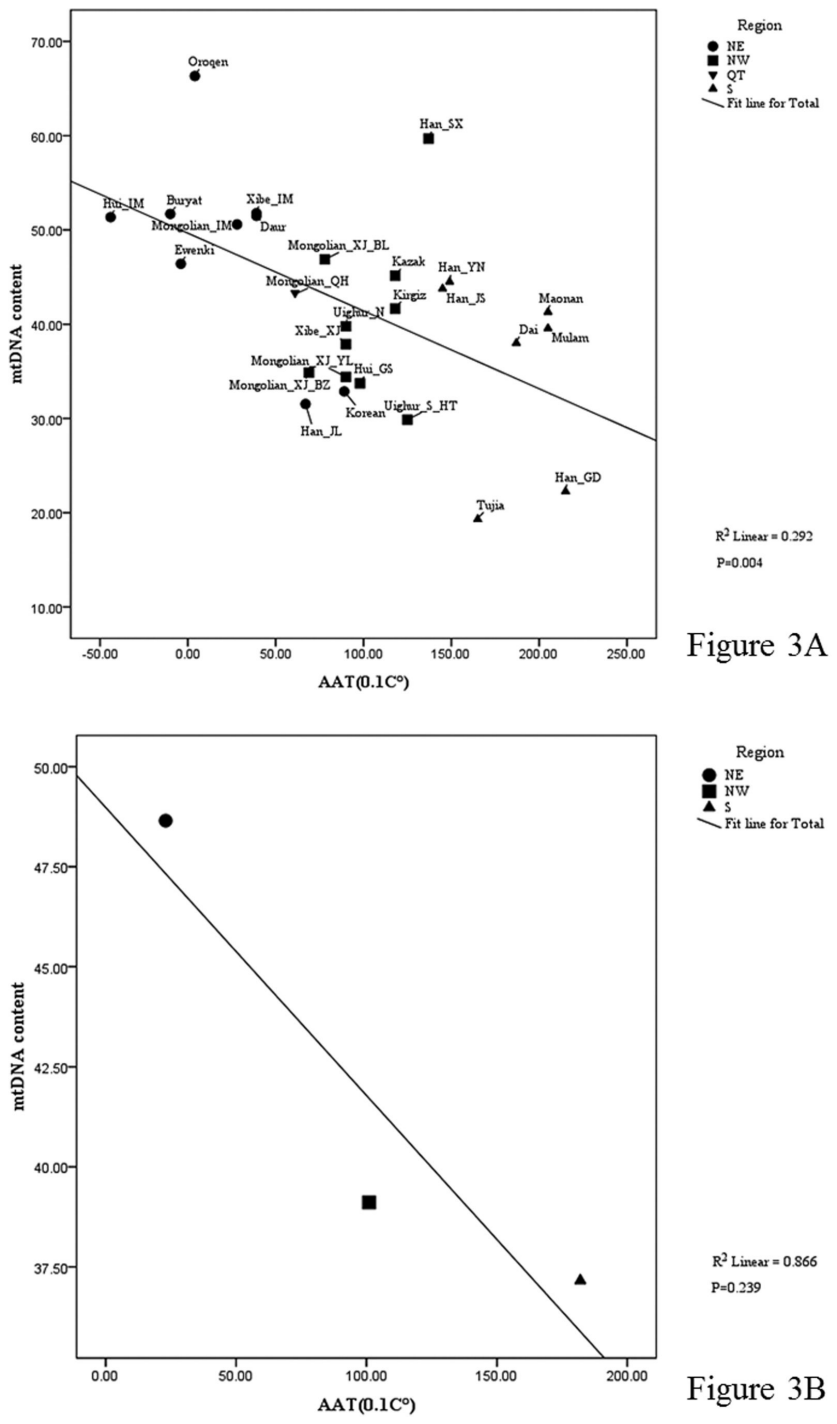
Correlation of mtDNA content with temperature factors in Chinese populations. (A) The results of regression analysis between temperature and mtDNA content in each subpopulation (R^2^=0.292, *P*=0.4×10^-2^); (B) the regression between temperature and mtDNA content considering 3 geographical clusters (R^2^=0.866). (NE, the northeastern China; NW, the northwestern China; S, the southern China; QT, Qinghai-Tibet plateau). .

Taking into account the important role of mtDNA content in regulating the human bioenergy processes [10-29], it is plausible that environmental factors, especially climate, are the driving factors shaping the distribution of the mtDNA copy number. To test whether a correlation between mtDNA content and climate can be detected, we collected climate information for the location of each sampled population (Table 1). Regression analysis showed a significant association between the distribution pattern of mtDNA content and temperature factors (R^2^_AAT_=0.292, *P*_AAT_=0.4×10^-2^; R^2^_AATmax_=0.289, *P*_AATmax_=0.4×10^-2^; R^2^_AATmin_=0.293, *P*_AATmin_=0.4×10^-2^; Figure 3a and Figure S1). Further, populations from the same geographic region (with similar temperature), even belonging to different ethnic groups, tend to share similar level of mtDNA content (R^2^_AAT_=0.866; Table 2 and Figure 3b). 

**Table 2:** mtDNA content level (minimum\median\maximum\mean) and climatic information for the 3 groups classified by their geographical locations.

**Region**	**Sample size**	**Minimum**	**Median**	**Maximum**	**Mean**	**AAAP^a^**	**AAT^b^**	**AATmax^c^**	**AATmin^d^**	**AARH^e^**	**ASH^f^**
NE^g^	320	4.89	41.37	166.91	48.65	9594	23	87	-34	64	27288
NW^h^	593	4.39	32.28	270.22	39.11	9146	101	167	43	59	25744
S^i^	282	2.56	40.58	106.83	37.16	9522	182	230	146	75	17939

a. AAAP, annual average atmospheric pressure; b. AAT, the annual average temperature; c. AATmax, the average maximum temperature; d. AATmin, the average minimum temperature; e. AARH, annual average relative humidity; f. ASH, annual sunshine hour; g. NE, northeastern China; h. NW, northwestern China; i. S, southern China.

**Figure 4:**
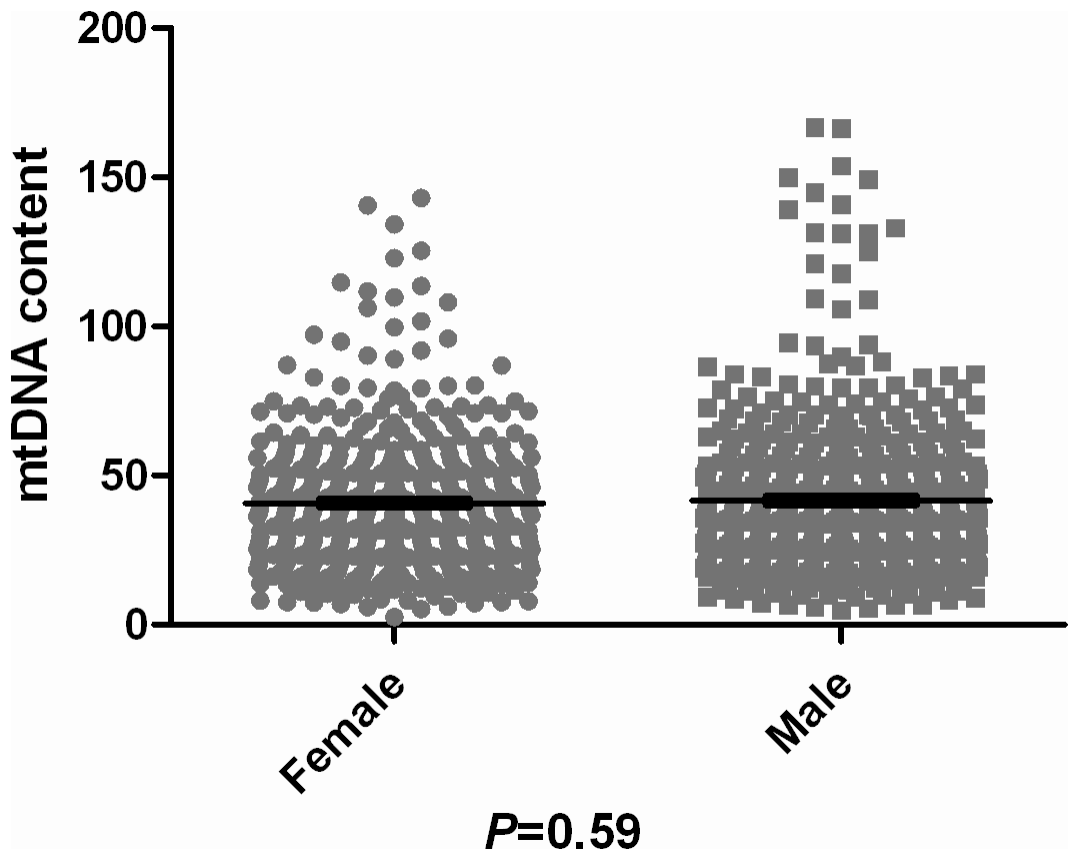
mtDNA content in males and females. Taking into account the sex and age of all samples for which this information available, the mean mtDNA content is 40.79 for the female group (432 individuals) while it is 41.67 for the male group (493 individuals) (*P*_female/male_=0.59).

**Figure 5:**
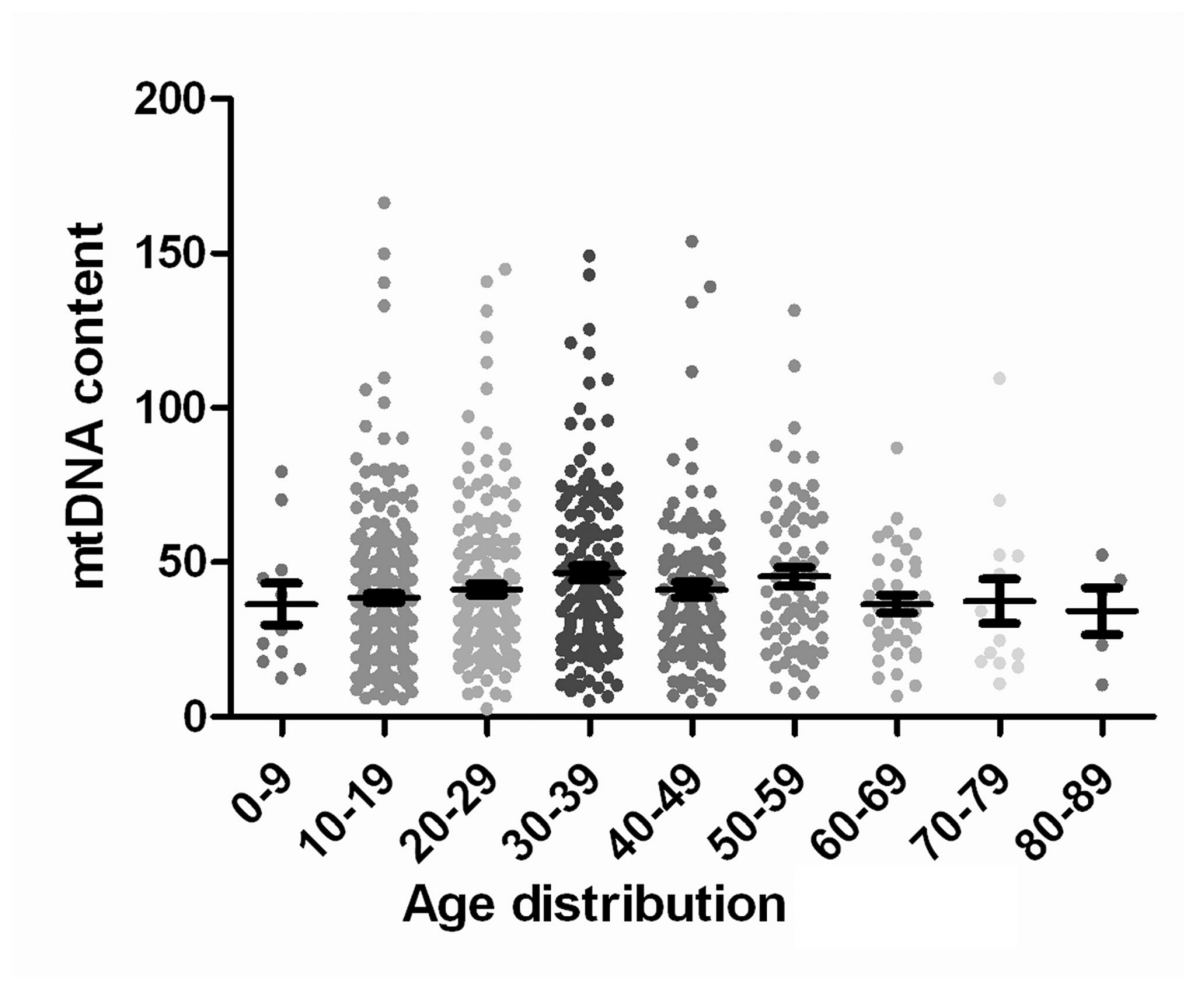
The distribution of mtDNA content in different age groups. Upon dividing the 851 samples with available age information into 9 groups by 10 year intervals, mtDNA content reaches a peak in the group of 30-39 years, but no significant difference among the age groups is observed (Kruskal-Wallis test; *P*=0.09).

To evaluate whether the observed significant difference in the mtDNA content between the NE and S groups is stable, sex and age information of the individuals belonging to both groups were retrieved and their influence on the result was assessed. As shown in Table S1, the mtDNA content of NE populations is still larger than S populations after the samples are divided into male and female sub-groups (*P*_male_=1×10^-3^ and *P*_female_=0.05). Likewise, a nonparametric test revealed no significant difference between NE and S groups in their age distribution (*P*=0.74). Taken together, it seems unlikely that the observed distribution pattern of mtDNA content among the populations can be simply attributed to the influence of sex or age. Indeed, when taking into account all the studied samples with sex and age information available, the mean mtDNA content of the female group (consisting of 432 females) is 40.79 while for the male group (with 493 males) it is 41.67 (*P*_female/male_=0.59; Figure 4). Upon dividing After the 851 samples (with available age information retrieved) being divided into 9 groups of 10 year intervals, the mtDNA content reaches a peak in the group of 30-39 years, but no significant difference among the age groups is observed either (Kruskal-Wallis test; *P*=0.09; Figure 5).

Although it is recognized that mtDNA serves as an important factor in bioenergy and thermogenesis and is thus may be involved in human adaptation [10], conflicting results were obtained regarding the association between mtDNA haplogroups and climate adaptation [9-14], thus invoking some doubt as to the role of mtDNA in human adaptation. By collecting and analyzing the distribution patterns of the mtDNA content among 27 Chinese populations residing in different geographic regions across China, our study reveals a significant association between variation in mtDNA content and climate, which remains even after other factors such as age and sex were considered. Therefore, it seems that the genetic imprints of human adaptation to different geographic areas with variable climate conditions can be detected on the mtDNA copy number instead of mutations (by way of haplogroups).

Indeed, the mtDNA content can affect the expression level of energy metabolism enzymes, such as those involved in ATP synthesis [30]. mtDNA copy number is positively associated with the expression of mitochondrial genes [31] and lower mtDNA content with the down-regulation of enzyme activities [17,32]. Rather than the result of a random process, the significantly higher mtDNA content in northern Chinese populations may confer some selective advantages in adapting to a climate with much lower temperature, since high mtDNA content can increase the expression of energy metabolism enzymes and thus may accelerate thermogenesis.

In summary, our present finding is that the mtDNA content, instead of mtDNA haplogroup, may contribute to the adaptation of human populations to different climate environments. The association between mtDNA content and the expression level of energy metabolism enzymes is in accordance with the hypothesis that mtDNA content has played an important role in regulating the balance between energy production and thermogenesis, with higher expression beneficial in colder environments.

## Materials and Methods

### Sampling

Peripheral blood samples were collected with informed consent from 1207 Chinese individuals in the present study, representing 27 Chinese ethnic populations residing in five climate zones of China (Table 1). Climate information for each population was obtained from the China Meteorological Data Sharing Service System [33], including annual average atmospheric pressure (AAAP), annual average temperature (AAT), annual average maximum temperature (AAT_max_), annual average minimum temperature (AAT_min_), annual average relative humidity (AARH) and annual sunshine hour (ASH) (Table 1 and Figure S1). For each sample, genomic DNA was extracted by the phenol/chloroform extraction method. Each participant was informed about the study and signed a consent form. This project was approved by the Ethics Committee at Kunming Institute of Zoology, Chinese Academy of Sciences.

### Quantitative PCR

Quantitative PCR was performed to measure the copy number of mtDNA using SYBR® Premix ExTaqTM II and Perfect Real-time PCR? (Takara Bio Inc., Shiga, Japan) on a Bio-Rad IQ5 Muilt-color Real-time PCR Detection System (Bio-Rad, Hercules, CA). The primers L394 and H475, which amplify a D-loop region of the mtDNA, were used for measuring the mtDNA copy number. The primers HBG1 and HBG2 were employed to amplify the globulin gene as a standard across samples [33]. The procedure for using real-time PCR to evaluate mtDNA content has been described elsewhere [34,35]. To ensure data quality, samples with a standard deviation greater than 0.5 across replicates were excluded.

### Data analyses

Individuals were divided into groups based on the sample location. A Kruskal-Wallis test was applied to calculate the difference between the subpopulations. PCA and canonical correlation analysis were used to identify the influence of mtDNA lineage. All tests were carried out using SPSS v16.0 (SPSS Inc., Chicago, IL). The distribution figures were drawn using Prism 5 (GraphPad Software, Inc., CA) and Surfer v8.0 (Golden Software, Inc., CO). The mtDNA hypervariable segment I sequences data were obtained from previous studies [20-28]. The sequences were then edited and aligned using the DNASTAR v5 software (DNAStar Inc., Madison, Wisc.), and mutations were scored relative to the revised Cambridge reference sequence (rCRS) [36].

## Supporting Information

Figure S1 **The correlation between mtDNA content and average maximum temperature (AATmax) and average minimum temperature (AATmin).** Circle, square, triangle, and inverse triangle indicate northeastern (NE), northwestern (NW), southern (S), and Qinghai-Tibet plateau (QT) populations.
(TIF)

Figure S2 **The correlation between mtDNA content and annual average atmospheric pressure (AAAP), annual average relative humidity (AARH), and annual sunshine hour (ASH).** Circle, square, triangle, and inverse triangle indicate northeastern (NE), northwestern (NW), southern (S), and Qinghai-Tibet plateau (QT) populations, respectively.
(TIF)

Materials S1 **Supporting information references.**
(DOC)

Table S1 **The mtDNA content between NE and S groups by different gender sub-groups.**
(XLS)

Table S2 **Information of samples with mtDNA control region data available.**
(XLS)
