# Supplementary material for: Mitochondrial DNA Content Contributes to Climate Adaptation Using Chinese Populations as a Model
Source: PLoS One. 2013 Nov 8;8(11):e79536. doi: 10.1371/journal.pone.0079536 (PMC3821843; doi:10.1371/journal.pone.0079536)
Supplement: Materials S1 — Supporting information references. (DOC) [file pone.0079536.s003.doc]

**Supplemental Material**

Figure S1. The correlation between mtDNA content and average maximum temperature (AATmax) and average minimum temperature (AATmin). Circle, square, triangle, and inverse triangle indicate northeastern (NE), northwestern (NW), southern (S), and Qinghai-Tibet plateau (QT) populations.

Figure S2. The correlation between mtDNA content and annual average atmospheric pressure (AAAP), annual average relative humidity (AARH), and annual sunshine hour (ASH). Circle, square, triangle, and inverse triangle indicate northeastern (NE), northwestern (NW), southern (S), and Qinghai-Tibet plateau (QT) populations, respectively.

Table S1. The mtDNA content between NE and S groups by different gender sub-groups.

Table S2. Information of samples with mtDNA control region data available.

**References**

Kong, Q. P., Y. G. Yao, M. Liu, S. P. Shen, C. Chen, C. L. Zhu, M. G. Palanichamy, and Y. P. Zhang. 2003. Mitochondrial DNA sequence polymorphisms of five ethnic populations from northern China. Hum Genet **113**:391-405.

Li, H., X. Cai, E. R. Winograd-Cort, B. Wen, X. Cheng, Z. Qin, W. Liu, Y. Liu, S. Pan, J. Qian, C. C. Tan, and L. Jin. 2007. Mitochondrial DNA diversity and population differentiation in southern East Asia. Am J Phys Anthropol **134**:481-488.

Wang, W. Z., C. Y. Wang, Y. T. Cheng, A. L. Xu, C. L. Zhu, S. F. Wu, Q. P. Kong, and Y. P. Zhang. 2010. Tracing the origins of Hakka and Chaoshanese by mitochondrial DNA analysis. American Journal of Physical Anthropology **141**:124-130.

Wen, B., H. Li, D. Lu, X. Song, F. Zhang, Y. He, F. Li, Y. Gao, X. Mao, and L. Zhang. 2004a. Genetic evidence supports demic diffusion of Han culture. Nature **431**:302-305.

Wen, B., X. Xie, S. Gao, H. Li, H. Shi, X. Song, T. Qian, C. Xiao, J. Jin, B. Su, D. Lu, R. Chakraborty, and L. Jin. 2004b. Analyses of genetic structure of Tibeto-Burman populations reveals sex-biased admixture in southern Tibeto-Burmans. Am J Hum Genet **74**:856-865.

Yao, Y. G., Q. P. Kong, H. J. Bandelt, T. Kivisild, and Y. P. Zhang. 2002a. Phylogeographic differentiation of mitochondrial DNA in Han Chinese. Am J Hum Genet **70**:635-651.

Yao, Y. G., Q. P. Kong, C. Y. Wang, C. L. Zhu, and Y. P. Zhang. 2004. Different matrilineal contributions to genetic structure of ethnic groups in the silk road region in china. Mol Biol Evol **21**:2265-2280.

Yao, Y. G., L. Nie, H. Harpending, Y. X. Fu, Z. G. Yuan, and Y. P. Zhang. 2002b. Genetic relationship of Chinese ethnic populations revealed by mtDNA sequence diversity. American Journal of Physical Anthropology **118**:63-76.

Zhang, Y., Q. Xu, H. Cui, Y. Cui, H. Lin, K. Kim, and J. Lee. 2005. Haplotype diversity in mitochondrial DNA hypervariable region I, II and III in a Korean ethnic group from northeast China. Forensic science international **151**:299-301.
